# Phylogenetic assemblage structure of North American trees is more strongly shaped by glacial–interglacial climate variability in gymnosperms than in angiosperms

**DOI:** 10.1002/ece3.2100

**Published:** 2016-04-03

**Authors:** Ziyu Ma, Brody Sandel, Jens‐Christian Svenning

**Affiliations:** ^1^ Section for Ecoinformatics and BiodiversityDepartment of BioscienceAarhus UniversityNy Munkegade 114DK‐8000Aarhus CDenmark

**Keywords:** Gymnosperm, Net Relatedness Index, paleoclimate, phylogenetic diversity, phylogenetic endemism, tropical niche conservatism

## Abstract

How fast does biodiversity respond to climate change? The relationship of past and current climate with phylogenetic assemblage structure helps us to understand this question. Studies of angiosperm tree diversity in North America have already suggested effects of current water–energy balance and tropical niche conservatism. However, the role of glacial–interglacial climate variability remains to be determined, and little is known about any of these relationships for gymnosperms. Moreover, phylogenetic endemism, the concentration of unique lineages in restricted ranges, may also be related to glacial–interglacial climate variability and needs more attention. We used a refined phylogeny of both angiosperms and gymnosperms to map phylogenetic diversity, clustering and endemism of North American trees in 100‐km grid cells, and climate change velocity since Last Glacial Maximum together with postglacial accessibility to recolonization to quantify glacial–interglacial climate variability. We found: (1) Current climate is the dominant factor explaining the overall patterns, with more clustered angiosperm assemblages toward lower temperature, consistent with tropical niche conservatism. (2) Long‐term climate stability is associated with higher angiosperm endemism, while higher postglacial accessibility is linked to to more phylogenetic clustering and endemism in gymnosperms. (3) Factors linked to glacial–interglacial climate change have stronger effects on gymnosperms than on angiosperms. These results suggest that paleoclimate legacies supplement current climate in shaping phylogenetic patterns in North American trees, and especially so for gymnosperms.

## Introduction

Climate change strongly influences biodiversity by changing species ranges (Davis and Shaw 2001) as well as species composition (Williams et al. 2004) and species richness of communities (Currie et al. 2004). An important question is whether biological responses to climate change are generally slow or fast relative to the changes themselves. If they are fast, then most ecological patterns should be understandable by considering only current conditions. On the other hand, if they are slow, then climate changes in the past could have potentially long‐lasting consequences for ecological patterns (Svenning et al. 2015). While current climate can directly regulate biodiversity via ecological limits set by the water–energy balance (Currie et al. 2004), paleoclimate can impact migration, speciation, and extinction via periodic transitions from warm to cold phases (Dynesius and Jansson 2000; Jansson and Dynesius 2002). As a result, the biodiversity patterns observed at the current time may be also influenced by climate conditions in the past (Eiserhardt et al. 2015b). Thus, in the fast‐changing climate of the Anthropocene, biodiversity pattern studies, conservation management, and planning should rely on more explicit determination of the time lags involved in the biodiversity–climate relationships (Dullinger et al. 2012; Wolkovich et al. 2014).

Phylogenetic assemblage structure is an important aspect of biodiversity. It combines both ecological processes and evolutionary histories and is therefore inherently linked to both current and past environmental conditions (Cavender‐Bares et al. 2009). Current environment may limit phylogenetic diversity, if the functional traits are, to some extent, conserved on the phylogeny (Flynn et al. 2011). In some cases, species assemblages are composed of species that are more closely related than a random sample from the available species pool, causing phylogenetic clustering, versus phylogenetic overdispersion in the opposite case. Phylogenetic clustering can be generated by environmental filtering. For example, the absence of certain lineages outside of the tropics leads to phylogenetic clustering (Eiserhardt et al. 2015a). Biotic factors may also have effects; for example, competition may result in phylogenetic overdispersion (Cavender‐Bares et al. 2004). Past climatic conditions can also leave a long‐term imprint on phylogenetic diversity and clustering patterns (Kissling et al. 2012), due to their influence on clade‐specific speciation, extinction, and migration rates. For example, historical area of habitats can be a strong predictor of diversification (Jetz and Fine 2012) and glacial–interglacial climate variability can impose recolonization limits to particular lineages and cause clade‐specific extinctions (Svenning 2003; Eiserhardt et al. 2015a). In addition, range contractions, extinctions, and clade‐specific diversification in restricted areas caused spatial restriction of unique lineages, or endemism, which can also be strongly shaped by long‐term climate stability (Jansson 2003). On a global scale, it has been shown that high endemism is associated with high long‐term climate stability (Sandel et al. 2011). Such patterns could also affect phylogenetic assemblage structure as they are shaped by extinction and/or speciation patterns (Dynesius and Jansson 2000; Jansson and Dynesius 2002).

A prominent explanation of the global biodiversity gradient is the tropical niche conservatism (TNC) hypothesis, which reasons that the predominant tropical climate in Earth's history, the tendency for phylogenetic conservation of ecological traits, and the limited dispersal and survival of species out of the tropics has resulted in less diverse species assemblages in higher latitudes (Wiens and Donoghue 2004). The TNC hypothesis predicts that the origin of temperate species can be traced to the tropics, with ancestral states of their adaptive traits (such as temperature tolerances) also being tropical (Kerkhoff et al. 2014), that more phylogenetic clustering is observed toward more limiting environmental factors (Qian et al. 2013), and that the temperate clades are younger and nested within tropical clades (Hawkins et al. 2014).

Disequilibrium dynamics between biodiversity and climate may affect large biogeographic regions (Svenning and Sandel 2013). In European forests, the influence of Last Glacial Maximum (LGM) climate on tree species richness is still apparent (Svenning and Skov 2007) and limited postglacial migration has prevented many tree species from fully filling their suitable range, as defined by current climate (Normand et al. 2011). North America hosts a wide range of forest biomes, from vast boreal and temperate forests in the north to subtropical and tropical forest patches in the south, and a diverse tree flora, with 679 species occurring north of the United States–Mexican border (Little 1971; Tucker 1983). Species richness is strongly correlated with current climate and can be explained mainly by the energy–water balance (Currie et al. 2004). Evolutionary factors appear to influence the pattern as well (Qian et al. 2015). However, the continent was extensively glaciated at the LGM (Peltier 1994) with compositionally different forest biomes existing at that time (Williams et al. 2000; Strong and Hills 2005) in multiple scattered forest refugia (Anderson et al. 2006; Gonzales et al. 2008). In addition, many trees in North America may not be able to adjust to the pace of contemporary climate change (Zhu et al. 2012). Therefore, exploring the relationship between current and past climate and phylogenetic assemblage structure of North American forests could be important for elucidating if tree diversity patterns here are shaped by glacial–interglacial disequilibrium dynamics and also for testing the TNC hypothesis.

Recent studies addressing the phylogenetic patterns in North American forests have focused on angiosperm trees only (Hawkins et al. 2014; Kerkhoff et al. 2014; Qian et al. 2015). However, a great share of the North American forests is dominated by gymnosperms, and 118 of 679 species in the tree flora are gymnosperms (Little 1971; Tucker 1983). The extant gymnosperms are mostly the result of diversification from long branches in their phylogeny, otherwise strongly pruned by Cenozoic extinctions, with some isolated relict species also remaining (Crisp and Cook 2011). Ecologically, they are generally less competitive than angiosperms in warm and wet environments, but relatively more persistent in environments with stressful conditions for plant growth (Bond 1989; Brodribb et al. 2012). Hence, they may not show the same diversity gradients or the same responses to diversity‐influencing mechanisms as angiosperms, and might show even more disequilibrium with current climate because of particularly severe past extinctions and range contractions (Crisp and Cook 2011).

In this study, we used a well‐resolved supertree to map phylogenetic assemblage patterns of gymnosperm and angiosperm trees in North America to assess the relative roles of current climate, glacial climate, and topography in shaping these patterns. The phylogenetic assemblage patterns were quantified via three metrics. (1) Phylogenetic diversity (PD) was measured by summing diversification time of all lineages in a species assemblage (Faith 1992). (2) Phylogenetic clustering was represented by the Net Relatedness Index (NRI), which describes the relative relatedness between species pairs in an assemblage compared to a random null (Webb et al. 2002). (3) Phylogenetic endemism (PE) was derived from PD and range sizes of every branch in the phylogeny (Rosauer et al. 2009).

**Figure 1:**
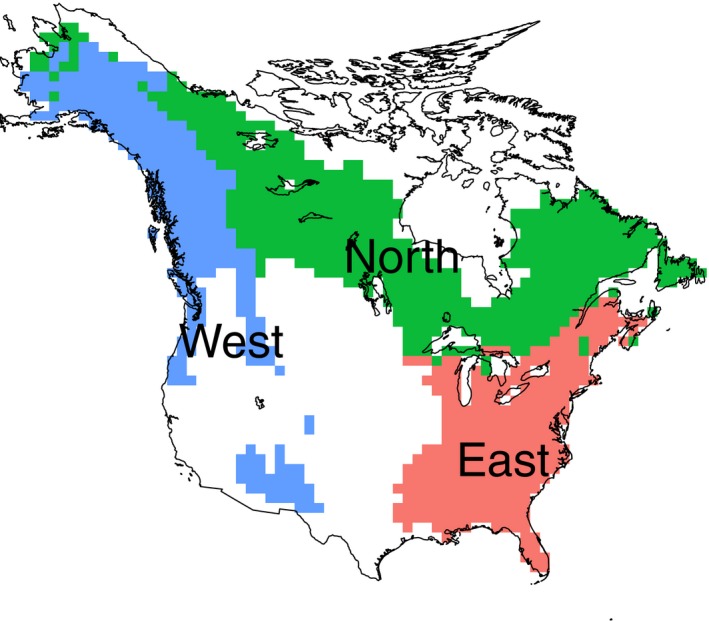
The forest regions were defined as combinations of CEC (Commission for Environmental Cooperation, 1997) Level I ecoregions of North America: The North (green) included the Taiga and Northern Forests; the East (red) included the Eastern Temperate Forests and Tropical Humid Forests; the West (blue) included the Marine West Coast Forests, Northwestern Forested Mountains, and Temperate Sierras; and the nonforest region (white) included all other ecoregion classifications.

Given the deep evolutionary separation between angiosperms and gymnosperms, the two groups were analyzed separately and the results were compared in parallel. Furthermore, as there could be region‐specific dynamics reflecting differences in regional environmental history and regional biogeographic patterns, we analyzed the relations both for North America overall and for the three major North American forest regions, separately (Fig. 1).

We focused on the following hypotheses:

1. Current climate is the dominant explanatory factor for phylogenetic assemblage structure of trees in North America, with temperature playing the most important role, so higher diversity and endemism is expected in warmer areas (Hawkins et al. 2003). Following the Tropical Conservatism Hypothesis (Hawkins et al. 2014), we also predict increasing phylogenetic clustering with decreasing temperature as well as with decreasing precipitation. We note that relations to current climate may in fact reflect relations to correlated deeper‐time climate patterns (Eiserhardt et al. 2015b).
2. Higher phylogenetic diversity and endemism occurs in areas less affected by glacial–interglacial climate instability and glaciation, that is, in areas with more stable climate (Sandel et al. 2011) or more heterogeneous topography, as this buffers against climate change (Ackerly et al. 2010), as well as in areas more accessible to postglacial recolonization. We note that topography may also influence assemblage structure via a contemporary habitat diversity effect (Kerr and Packer 1997; Anderson and Ferree 2010).
3. Gymnosperms, in competitive disadvantage with angiosperms in warm and wet conditions (Brodribb et al. 2012) and having a divergent evolutionary history strongly shaped by Cenozoic extinctions (Crisp and Cook 2011), will show different phylogenetic assemblage structure patterns from angiosperms and respond less to current, but more to past climate conditions.

## Materials and Methods

### Species distribution data

Digitized maps compiled from the Atlas of United States Trees were downloaded from the USGS Geosciences and Environmental Change Science Center (http://esp.cr.usgs.gov/data/little/). These maps include 679 species of native woody plants that can reach tree size, defined as at least 3 inches (76.2 mm) in diameter at breast height and 13 feet (nearly 4 m) in height (Little 1971). Maps were expert drawn to illustrate the natural distribution of tree species exclusive of changes caused by human disturbance following European settlement, such as deforestation.

Species distribution maps were rasterized in R 3.1.0 using the “maptools” and “raster” packages (Bivand et al. 2015b; Hijmans et al. 2015a) and mapped in Albers Equal Area projection with 100 × 100 km grid spanning the study area. The study area was defined as continental North America north of the US‐Mexico border, not including grid cells with more than 50% water surface. Taxonomic statuses were revised according to The Plant List (http://www.theplantlist.org/), and taxa treated as synonyms in the maps had their distribution combined (see, Table S1 for detailed taxonomic revisions). In total, the study area consisted of 2671 grid cells and hosted 619 species, including 98 gymnosperms and 521 angiosperms.

### Phylogenetic analysis

The phylogenetic relationships of 98 gymnosperm species in this study were based on a dated tree of 489 extant conifers (Leslie et al. 2012). Five taxa in the Atlas of United States Trees were absent from this reference phylogeny, they were manually added to the tree with random branch lengths based on known phylogenetic relationships according to the Flora of North America (Flora of North America Editorial Committee, 1993), and the resulting phylogeny was attached in Supporting Informations.

For the 521 angiosperm species in this study, the reference phylogeny was the dated tree of the world's woody angiosperm species (Zanne et al. 2014). After resolving synonyms according to TPL, there were still 133 species in this study that were not included in the reference phylogeny, but all the families in the data set were covered by Zanne et al. (2014). These extra species were thus manually added as random branches in the clades representing their families from the reference phylogeny, or as random branches in the genus clades if congeneric species were present in the reference phylogeny. The final angiosperm phylogeny used for this study is available in Supplementary Materials. While this phylogeny is not well resolved near the tips, most of the structure at the family level is clear, so it should provide an adequate representation of deeper phylogenetic relations. The phylogenic trees were then converted into a matrix of pairwise divergence times between species using the “ape” R package (Paradis et al. 2004).

Phylogenetic structure was analyzed for the assemblages of species occurring in each 100 × 100 km^2^ cell for angiosperms and gymnosperms, separately. The three metrics were defined as follows: For a given grid cell, PD was the sum phyletic branch lengths involving all occurring taxa (eq. (1)), without considering the root branch length (Faith 1992); hence, cells with fewer than two species will not have a PD value. PE (eq. 2) was different from PD in weighting the branch lengths with their range in grid cells, with PE derived as the weighted sum (Rosauer et al. 2009)

(1) $$\mathrm{PD}=\sum_{c\in C}L_{c}$$

(2) $$\mathrm{PE}=\sum_{c\in C}\frac{L_{c}}{R_{c}}$$

where *C* is a phylogenetic clade spanned by minimal branches to join all taxa in each given grid cell, and *c* is any branch between two nodes within *C*. *L*_*c*_ is the length of branch *c*, and *R*_*c*_ is its range, defined as numbers of grid cells where branch *c* occurred.

Net Relatedness Index was calculated from Mean Pairwise Distance (MPD), the mean of phyletic branch length distances between all pairs of species occurring in a grid cell, and then the effect size was standardized by the mean and standard deviation of MPD values calculated from 100 null models that draw random species of the same richness of the grid from the continental species pool (eq. (3)).

(3) $$\mathrm{NRI}=-1\times \frac{{\mathrm{MPD}}_{\mathrm{obs}}-\mathrm{mean}\left({\mathrm{MPD}}_{\exp}\right)}{\mathrm{sd}({\mathrm{MPD}}_{\exp})}$$

Where MPD_obs_ is calculated from species occurring in the given grid cell, and MPD_exp_ is the expected MPD distribution from the 100 random null models.

Phylogenetic diversity and NRI were derived from the “picante” R package (Kembel et al. 2010); note that NRI was multiplied by −1 in equation (3), so that positive NRI is associated with less MPD than expected mean (phylogenetic clustering). PE was calculated using the *phylo.endemism* function written by Nipperess (2012) in R 3.1.0. and was log‐transformed after adding 1 in later data analysis.

### Environmental measures

#### Current climate data

Mean annual temperature (MAT), minimum temperature of the coldest month (TMIN), temperature seasonality (TES), mean annual precipitation (MAP), and precipitation seasonality (PRS) were obtained from WorldClim (Hijmans et al. 2005). Then, the annual water balance (WBL) was calculated as WBL = MAP – PET, where PET is the annually summed potential evapotranspiration from the Global Aridity and PET Database (Zomer et al. 2008). All data were obtained in a resolution of 2.5 arcsec, then projected to Albers Equal Area projection and aggregated to the 100‐km grid.

#### Elevation model

A 2.5 arcsec Digital Elevation Model from the NASA Shuttle Radar Topography Mission (Farr et al. 2007) was projected to 5‐km resolution in Albers Equal Area projection, and the standard deviation of elevation in each 100‐km grid cell was used as a measure of elevation heterogeneity (EHET).

#### Paleoclimate data

Paleoclimate reconstructions were obtained from the MIROC‐ESM 2010 climate model (Watanabe et al. 2011), which included MAT, TES, MAP, and PRS estimates from the LGM, statistically downscaled and available from WorldClim (Hijmans et al. 2005). In addition, WBL at the LGM was calculated from the WorldClim monthly precipitation and temperature data using the method of Skov and Svenning (2004).

Two measures related to paleoclimate instability were applied. The first, climate change velocity, describes the rate of displacement of a climate isocline through time (Loarie et al. 2009). The chosen variable in this study was the velocity of temperature change (VT), which is the ratio of spatial versus temporal MAT change from LGM to now (Sandel et al. 2011). The velocity reflects the capacity of heterogeneous regions to buffer species against temperature change. It can be interpreted as the migration rate necessary to track a particular climatic condition.

The second measure of climate instability was postglacial tree accessibility (ACC), defined as the inverse distance of a pixel to the LGM distribution of forests. This distribution was estimated by hindcasting a model trained from the current distribution of forests. Land cover data in 2010 from NASA's MODIS (Moderate Resolution Imaging Spectroradiometer, NASA LP DAAC, 2001) were downloaded from the Global Land Cover Facility (GLCF) database in the University of Maryland (ftp://ftp.glcf.umd.edu/modis/). This was then converted to forest presence/absence by considering any grid cell with >25% tree cover to be forest.

Forest presence during the LGM was inferred from climate data using a Maximum Entropy Species Distribution Model (MaxEnt SDM) (Phillips et al. 2006). The MaxEnt SDM was built using present MAT, water balance (WBL; calculated using the method of Skov and Svenning (2004) due to the limited availability of LGM climate variables), TES, and PRS as predictors and the forest presence as response. A random quarter of the data was left out to test the model while the other 75% were used to train it. The SDM showed adequate performance (AUC train: 0.754, test: 0.938). The same set of climate data from 21 ka BP were then used as input to hindcast LGM forest presence. In order to convert the continuous MaxEnt SDM output to binary data, a threshold was applied using the method to equate entropy of thresholded and original distributions. The grid cells with LGM forest presence probability above the threshold were identified as the sources of postglacial tree migration. Calculations were performed in R with the “dismo” (Hijmans et al. 2015b) and “rJava” (Urbanek 2015) packages.

**Figure 2:**
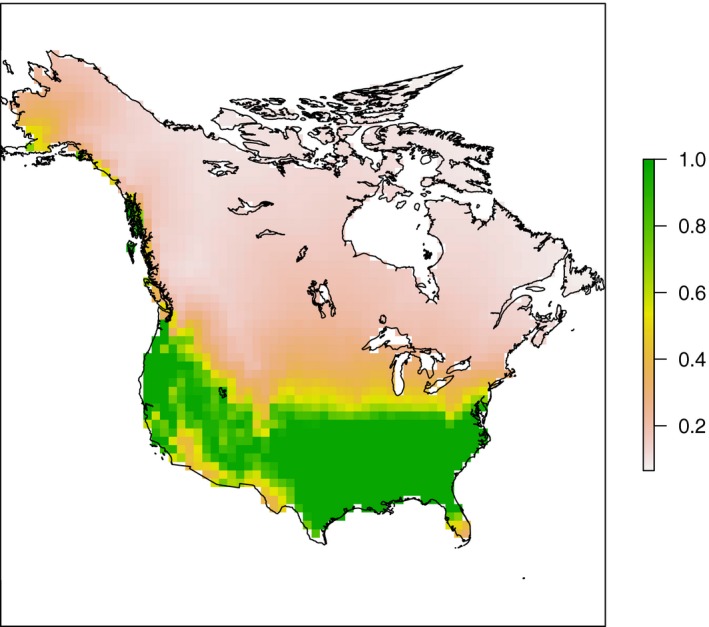
Accessibility, calculated by the reciprocal of cumulative cost distances to grids from in situ LGM forest presence, which was hindcasted using MaxEnt species distribution modeling. The cost of tree migration was calculated based on Euclidean distance of current climate.

Postglacial tree accessibility (ACC) was based on least cost paths from the estimated LGM distribution of forests. The cost distance between neighboring cells was based on their Euclidean distance in current climate space, calculated using the first three components of a principal component analysis including 19 current BioClim variables from WorldClim. Accumulated cost from each grid cell to all the sources was calculated using the “gdistance” package (Etten 2015) with the least cost route. ACC of each cell was then defined as the reciprocal of the accumulated cost distance plus 1, so that it ranges from 0 to 1, where 1 indicates in situ LGM forest presence and 0 indicates an infinite cost to disperse from the sources(Fig. 2).

### Data analysis

All predictors for current environment, climate change velocity, and postglacial accessibility were used to model tree diversity patterns in a multiple regression analysis. Thus, the predictors include eight continuous variables (MAT, TMIN, WBL, PRS, TES, ACC, VT, and EHET). ACC and VT were log‐transformed, while EHET was square‐root‐transformed to approximate normality in distribution. Pearson's correlation coefficients among the eight continuous predictor variables were calculated to assess collinearity (Table 1). Variables with high pairwise correlation (|*r*| > 0.5) were grouped, forming two groups of collinearity: one included MAT, TMIN, TES, and ACC; the other included VT and EHET. Only one variable from each group was used in the model selection process, resulting in eight “full model” formulas and their nested subsets.

**Table 1:** Pearson's correlation coefficient (*r*) of pairwise relationships between each response and predictor variables in the multiple regression models. Responses: phylogenetic diversity (PD), Net Relatedness Index (NRI), and phylogenetic endemism (PE) for angiosperms (A) and gymnosperms (G); Predictors: mean annul temperature (MAT), temperature of coldest month (TMIN), water balance (WBL), precipitation seasonality (PRS), temperature seasonality (TES), temperature change velocity since LGM (VT), tree accessibility (ACC), and elevation heterogeneity (EHET). Correlation among predictors was also assessed to identify possible collinear groups (|*r*| > 0.5, in bold)

Response	MAT	TMIN	WBL	TES	PRS	EHET	VT	ACC
PD.A	**0.783**	**0.745**	0.158	−**0.579**	−0.435	−0.236	0.235	**0.674**
NRI.A	−0.377	−0.388	0.086	0.330	0.029	0.175	−0.101	−0.357
PE.A	**0.868**	**0.868**	0.132	−**0.736**	−0.385	−0.084	0.097	**0.747**
PD.G	−0.043	0.011	0.433	−0.139	−0.263	0.186	0.038	−0.206
NRI.G	0.382	0.390	−0.197	−0.352	0.071	0.182	−0.224	0.450
PE.G	0.447	0.487	0.480	−0.535	−0.354	0.261	−0.130	0.293

Collinearity		TMIN	WBL	TES	PRS	EHET	VT	ACC
MAT		**0.960**	0.007	−**0.816**	−0.372	−0.107	0.157	**0.797**
TMIN			0.115	−**0.932**	−0.375	0.055	−0.015	**0.811**
WBL				−0.235	−0.413	0.098	−0.072	−0.028
TES					0.378	−0.272	0.190	−**0.703**
PRS						0.028	−0.174	−0.240
EHET							−**0.817**	−0.021
VT								−0.018

For each response variable (PD, NRI, and PE for angiosperms or gymnosperms), 120 candidate models were fitted, and candidate models were selected by comparing Akaike's information criterion (AIC). To correct spatial autocorrelation, simultaneous autoregressive models with spatial error (SARerr) were used, and all models built by possible combinations of predictors with ΔAIC values <10 from the model with lowest AIC were used in model averaging. Model‐averaged coefficients for the predictors were Akaike weighted means of the remaining models' standardized coefficients, and the importance of each predictor was defined as the sum of Akaike weights from models involving that predictor (Burnham and Anderson 2002). The spatial weight correction was derived from *k*‐neighbors method, and the value of *k* was picked decreasing from 10 to 4, until a Moran's I test of the SAR model showed no significant spatial autocorrelation (Kissling and Carl 2008). All statistical calculations were carried out in R 3.1.0. SAR models were built using the “spdep” package (Bivand et al. 2015a), and model selection was performed using the “MuMIn” package (Barton 2015).

In addition to the continental model, the analysis was also performed separately for three forest regions of North America (Fig. 1). Delineation of the forest regions was based on the Commission for Environmental Cooperation (CEC) Level I ecoregions, which divided North America into 15 broad ecological regions involving all major components of ecosystems – air, water, land, biota, and humans (Commission for Environmental Cooperation, 1997). In this study, the forested CEC Level I ecoregions north of the US–Mexican border were combined with the North (CEC Taiga and Northern Forests), the East (CEC Eastern Temperate Forests and Tropical Humid Forests), and the West (CEC Marine West Coast Forests, Northwestern Forested Mountains, and Temperate Sierras). For angiosperms, only a subset of the grid cells containing five or more species was used, as phylogenetic diversity measures for small sets of species can be noisy. Limiting the grids to the same standard for gymnosperms, lowered the sample size too much, grid cells with two or more species were all used in the analysis.

## Results

### Geographic patterns

**Figure 3:**
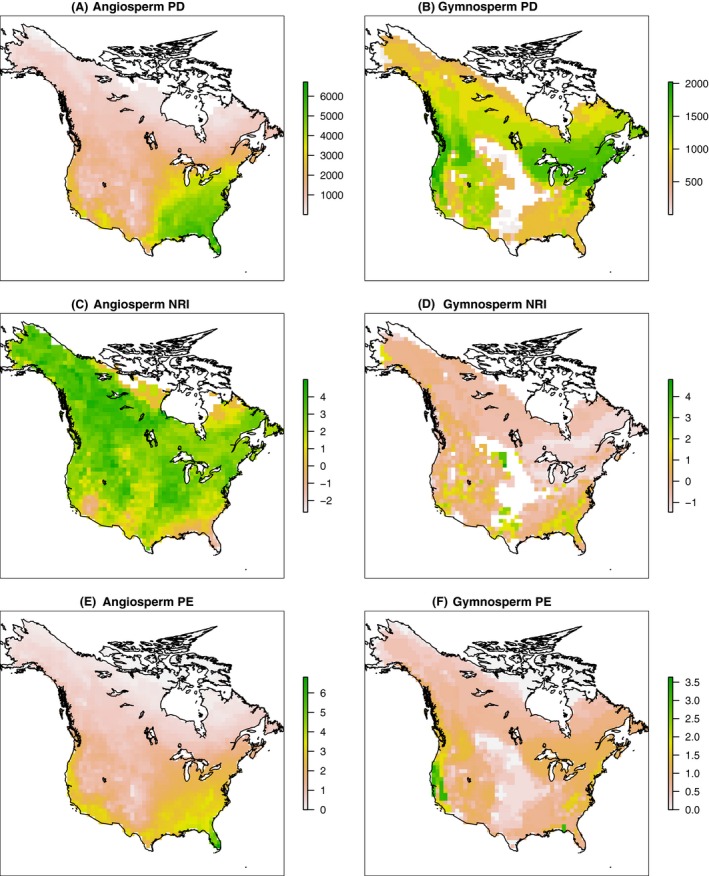
Phylogenetic diversity (PD) for angiosperms (A)/gymnosperms (B); Net Relatedness Index (NRI) measures for angiosperms (C)/gymnosperms (D); natural logarithm of phylogenetic endemism (PE) measures for angiosperms (E)/gymnosperms (F) in the study area of North America.

Angiosperm tree PD was highest in the southeastern quarter of North America, particularly in the eastern temperate to subtropical region, decreasing toward northern temperate and boreal forests or toward the central Great Plains, with the least diverse areas being the arctic and the deserts (Fig. 3A). The PD of gymnosperm trees did not follow the same pattern, as the northern temperate forests and mountain forests were more diverse than the boreal or subtropical forests, with several hot spots exhibiting high PD in the Pacific Coast Ranges (Fig. 3B).

With respect to NRI, angiosperm trees exhibited a general pattern of phylogenetic clustering, especially so in the boreal forests. Clustering became less pronounced toward the south and east, culminating with strong phylogenetic overdispersion in southern Florida. Regions with low tree species richness varied in their degree of phylogenetic clustering; for example, the arctic tundra was more overdispersed than the boreal forests and phylogenetic patterns of the Great Plains and deserts appeared stochastic (Fig. 3C).

In contrast to angiosperms, gymnosperms trees were mostly overdispersed, particularly in regions dominated by them, such as the boreal zone and the Pacific Northwest. Phylogenetic clustering occurred in the Appalachian Mountains, in the southeastern *Pinus* spp.‐dominated forests, in *Sequoiadendron giganteum* forests in California, in the Rocky Mountains, west of the Alaska Range where only two *Picea* spp. dominate, and in scattered arid spots where only a few *Juniperus* spp. survive (Fig. 3D).

Phylogenetic endemism showed similar patterns to PD. However, compared to PD, angiosperm PE varied more monotonically with latitudinal and water gradient, with higher endemism in the south and nonarid regions (Fig. 3E). In contrast, the pattern in gymnosperm trees corresponded less to latitude, and mountains appeared to host relatively high phylogenetic endemism. Isolated relict lineages such as *Sequoia sempervirens*,* Sequoiadendron giganteum*,* Torreya californica,* and *T. taxifolia* contributed strongly to hot spots of high endemism (Fig. 3F).

Factors related to the observed phylogenetic assemblage structures

- Temperature relations

**Figure 4:**
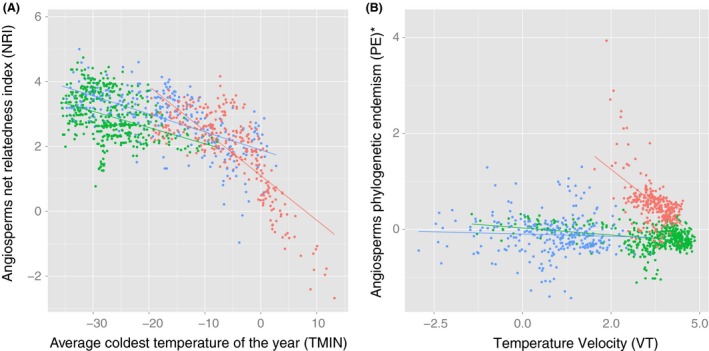
Bivariate plots explaining phylogenetic assemblage structure of angiosperm trees in North (green), West (blue), and East (red) forest regions. A: Net Relatedness Index (NRI) and current minimum temperature of the coldest month (TMIN), showing species occurring at locations with higher cold tolerance requirements, are more closely related than those occurring at warmer locations. B: phylogenetic endemism (PE*, partial residual controlling the effects of current climate) and postglacial climate change velocity in temperature (VT), showing higher endemism in climate stable regions. Data were extracted from grid cells of 100 × 100 km with five or more angiosperm tree species.

**Figure 5:**
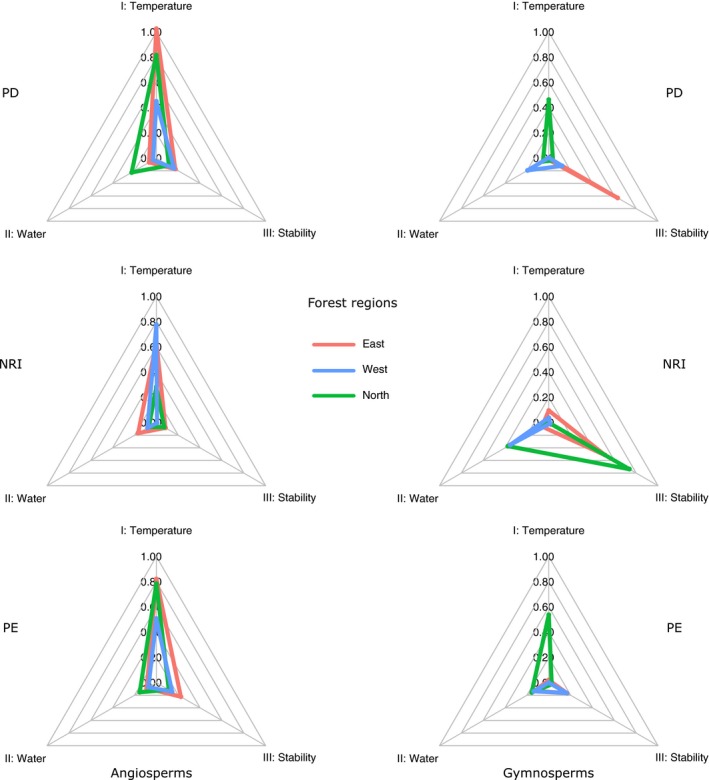
Differences in maximum effect sizes of predictor groups between angiosperms and gymnosperms in the models for the North, West, and East regions. Effect sizes are the magnitude of model‐averaged standardized coefficients. The predictors were grouped as follows: I. Temperature‐related: MAP, TMIN, and TES; II: Water‐related: WBL and PRS; III: Postglacial stability‐related: ACC, VT, and EHET. Angiosperms showed most response to current temperature, while gymnosperms were likely more influenced by postglacial climate stability.

Angiosperm tree PD and PE patterns in North America responded strongest to contemporary temperature. Mean annual temperature or coldest temperature showed significant positive correlations with diversity and endemism (Table 1). They also were selected as the most important predictors in continental PE model and in all the regional PD and PE models (Tables 2 and 3), where MAT or TMIN also had the largest average effect sizes (Fig. 5). NRI of angiosperm trees also responded mainly to current temperature, generally with increasing clustering with colder climate and higher TES (Tables 2 and 3). In particular, simple bivariate models showed strong negative effect of TMIN on NRI (Fig 4A).

**Table 2:** Multimodel inference from spatial‐error simultaneous autoregressive (SAR) models of phylogenetic diversity (PD), Net Relatedness Index (NRI), and phylogenetic endemism (PE) of angiosperms and gymnosperms in all grid cells covering continental North America north of the US–Mexican border

Model parameters	SAR‐averaged coefficients	Importance: sum Akaike weight	OLS *R* ^2^
Angiosperms PD			0.551
ACC	0.083***	0.993	
WBL	0.049***	1.000	
EHET	0.129***	1.000	
Angiosperms NRI			0.390
TMIN	−0.498***	0.997	
EHET	0.118***	0.999	
Angiosperms PE			0.779
MAT	0.097***	0.605	
WBL	0.097***	1.000	
PRS	0.033***	0.821	
EHET	0.139	1.000	
Gymnosperms PD			0.215
TMIN	−0.214**	0.810	
WBL	0.264***	1.000	
EHET	0.328***	1.000	
Gymnosperms NRI			0.271
ACC	0.108***	0.560	
WBL	−0.062***	0.680	
EHET	0.059***	0.633	
Gymnosperms PE			0.387
ACC	0.164***	0.958	
WBL	0.275***	0.995	
PRS	0.127***	1.000	
EHET	0.261***	1.000	

Only predictors with Akaike weights exceeding 0.5 are shown. Importance values and model‐averaged standardized parameters were reported. To describe explained variance, additional adjusted *R* ^2^ values were given for the ordinary least square (OLS) multiple regressions with the same formula of the lowest AIC SAR_err_ models.

Predictors include: MAT, mean annual temperature; TMIN, temperature of coldest month; WBL, water balance; PRS, precipitation seasonality; TES, temperature seasonality; VT, temperature change velocity since LGM; ACC, tree accessibility; EHET, elevation heterogeneity.

The significances of predictors in the lowest AIC models were given in the following scales: *** < 0.001 < ** < 0.01 < * < 0.05.

**Table 3:** Summaries of models predicting PD, NRI, and PE for the North, East, and West forest regions using temperature, water, and postglacial climate stability measures. Predictors were summarized in three groups: I. Temperature relations: MAT, TMIN, or TES; II. Water relations: WBL or PRS; III. Stability relations: ACC, VT, or EHET

	Angiosperms				Gymnosperms			
	Temperature	Water	Stability	*R* ^2^	Temperature	Water	Stability	*R* ^2^
PD North	**MAT 0.818**	**WBL** −**0.226**	EHET 0.115	0.76	**MAT 0.463**	WBL −0.012	VT −0.039	0.78
		**PRS** −**0.075**				**PRS** −**0.053**	EHET 0.024	
PD West	MAT 0.452	WBL –0.001	**EHET 0.162**	0.78	MAT 0.004	**WBL 0.194**	**EHET 0.122**	0.29
		**PRS 0.030**			TES 0.004	PRS 0.001		
PD East	**MAT 1.027**	WBL 0.019	**EHET 0.173**	0.85		WBL 0.096	**ACC** −**0.630**	0.76
		**PRS** −**0.067**				PRS −0.013	**EHET 0.188**	
NRI North	**MAT 0.284**	**WBL −0.069**	VT −0.072	0.29		**WBL 0.373**	**ACC 0.738**	0.36
	TES 0.038	PRS 0.023	**EHET 0.053**			**PRS 0.128**	EHET −0.008	
NRI West	**TMIN** −**0.777**	WBL 0.046	VT 0.006	0.46	TMIN −0.039	**WBL** −**0.354**	ACC −0.013	0.08
		**PRS** −**0.081**	EHET 0.004		TES −0.006	**PRS** −**0.161**	VT 0.011	
NRI East	**TES 0.636**	WBL −0.003	**VT 0.083**	0.76	MAT 0.096	**WBL 0.062**	**ACC 0.515**	0.44
		**PRS** −**0.167**				PRS −0.004	VT 0.023	
PE North	**MAT 0.785**	**WBL** −**0.152**	**EHET 0.114**	0.74	**MAT 0.538**	WBL 0.000	VT −0.014	0.78
		PRS −0.032				**PRS** −**0.156**	**EHET 0.027**	
PE West	**MAT 0.509**	**WBL 0.073**	**EHET 0.144**	0.82		**WBL 0.137**	**ACC 0.141**	0.49
		**PRS 0.056**				PRS 0.010	**EHET 0.165**	
PE East	**TMIN 0.822**	**WBL** −**0.088**	**VT** −**0.224**	0.91	TES −0.020	**WBL 0.128**	ACC −0.049	0.26
		PRS 0.022			MAT −0.011	PRS 0.001	**VT** −**0.168**	

Refer to Table 2 for abbreviations. Standardized coefficients of SAR_err_ models were presented, and they were averaged from multiple models by Akaike weight. Predictors' importance factors were also based on Akaike weight: A predictor was shown in bold with its importance > 0.5 and omitted with <0.1. In addition, *R* ^2^ values of the best OLS models were given to indicate the model's explanatory capability.

In contrast, gymnosperm trees showed less clear responses to current temperature. Continental model indicated that PD decreases with TMIN, and no strong temperature relationships were found for NRI and PE (Table 2). At regional extents, only the North showed a positive temperature effect on PD and PE (Table 3, Fig. 5).

Water relations

As for temperature, PD and PE patterns of both angiosperm and gymnosperm trees also responded to contemporary water relations. PD and PE generally increased with higher water balance in continental models; in addition, PRS also positively contributed to PE. Notably, the effect sizes of water relations for gymnosperms were always larger than those for angiosperms (Table 2). In regional models, water effects appeared much weaker and idiosyncratic. Angiosperm PD and PE decreased with water balance in the North, increased with seasonality in the West, while gymnosperm PD and PE increased with water balance in the East and West, and the effect sizes were greater than the water effects on angiosperms (Table 3, Fig. 5).

Higher water balance decreased phylogenetic clustering in gymnosperms, but showed no effects in angiosperms according to the continental models (Table 2). Water had important effects on gymnosperm NRI of the North and West regions: in the North, water balance and seasonality were positively associated with phylogenetic clustering, but these factors had negative effects in the West; that is, high precipitation and seasonality in the West led to phylogenetic overdispersion in gymnosperms.

Glacial‐interglacial climate stability relations

A consistent pattern existed for angiosperms and gymnosperms across the continent and all forest regions: Phylogenetic diversity and endemism were higher in locations with higher variability in elevation (Table 2) or lower glacial–interglacial temperature velocity (Table 3). High‐elevation heterogeneity was also associated with high phylogenetic clustering in gymnosperms (Table 2) and angiosperms in the North region (Table 3), while high velocity was associated with phylogenetic clustering of angiosperms in the East (Table 3).

The accessibility measure was highly correlated with all three current temperature variables, causing difficulty in estimating their separate effects (Table 1). Statistically, it predicted angiosperm PD, gymnosperm NRI, and PE better than the temperature variables in continental models. All the predicted variables respond positively to ACC with greater effect sizes for gymnosperms (Table 2). In regional models, only gymnosperms phylogenetic assemblage structure showed significant response to ACC (Fig. 5): In areas more accessible to forests after LGM, the models predicted gymnosperms to have less PD in the East, more PE in the West, and more phylogenetic clustering in the North and East (Table 3).

## Discussion

Phylogenetic assemblage structure in trees in North America exhibited relationships with both current and glacial–interglacial climate change. The former had the largest effects, but legacies of past climate change were also detectable and were especially important at regional extents and for the gymnosperms.

Phylogenetic assemblage patterns of North American tree species confirmed our first hypothesis, showing their alignment to the current climate gradient in both ecological function and evolutionary histories, especially in angiosperms. Temperature and water relations explained the main variations in phylogenetic diversity and endemism for angiosperm trees, following previously reported energy–water balance relations for species richness (Hawkins et al. 2003; Currie et al. 2004). Although this could suggest that there is only weak‐to‐moderate disequilibrium in North American angiosperm's response to climate, these current climate associations may be confounded with past climate effects on the biogeography of phylogenetic assemblage structures due to correlations between past and present geographic climate configurations (Eiserhardt et al. 2015b). The fact that phylogenetic clustering of angiosperms decreased almost monotonically with temperature (Fig. 4A) is consistent with an important influence of evolutionary history for the overall geographic patterning of the North American tree flora, shaped by cold tolerance arising only within a selection of clades nested within largely tropical groups, as argued by the TNC hypothesis (Qian et al. 2013; Kerkhoff et al. 2014). Gymnosperm trees also showed similar overall responses to warmer and wetter current climates, with more sensitivity to water relations compared to the angiosperms, but the models explained a smaller proportion of the variability in their phylogenetic assemblage structure and were less consistent among regions.

In addition to current climate, phylogenetic assemblage structure of North American trees also appeared to have consistent relations to glacial–interglacial climate stability, agreeing with our second hypothesis. In the continental model, phylogenetic diversity, clustering, and endemism all increased with EHET. This is consistent with mountain ranges acting as long‐term refugia (Steinbauer et al. 2013), buffering against selective extinctions during glacial–interglacial climate change (Eiserhardt et al. 2015a). Additionally, this is also consistent with the idea that, over long timescales, mountains produce biodiversity by simply having diverse substrates (Anderson and Ferree 2010) and steep climatic gradients that tend to promote population isolation and survival. In regional models, the results for the topographically flatter East and North forest regions suggested that this pattern was mainly a response to glacial–interglacial temperature change velocity (Fig. 4B), in line with global results linking long‐term climate stability to endemism (Jansson 2003; Sandel et al. 2011). Phylogenetic clustering in climatic stable areas should be reduced by the presence of multiple old lineages, for example, as possibly the case in Florida, where overdispersion of angiosperms was caused by diverse tropical taxa. However, stability may also promote recent diversification, so that overall clustering remained high, for example, in the southeast the presence of a *Pinus* species radiation associated with fairly stable climate conditions caused phylogenetic clustering, except where several Cupressaceae species also occurred (Eiserhardt et al. 2015b). Alternatively, assemblages in mountainous or low velocity areas generally had higher phylogenetic clustering, consistent with different clades surviving in different refugia and postglacial migration creating mixtures in recolonized nonrefuge areas; for example, *Picea glauca* might have migrated from an cryptic LGM refugium in Alaska (Anderson et al. 2006) to western Canada to become mixed with *Pinus contorta* immigrating from the south (Peteet 1991).

Gymnosperm trees did appear to be less responsive to the current climate than angiosperms, consistent with our third hypothesis. They showed positive responses in phylogenetic endemism and diversity to accessibility in the North but negative responses in the East. This pattern might be an outcome of several possible causes: First, gymnosperms may be more dispersal limited and may not have reached their potential range following the postglacial climate change. This idea is in agreement with pollen and macrofossil studies in western North America (Elias 2013). Notably, in the Pacific Northwest, all conifer species with the exception of *Pinus contorta* cannot establish in early succession, making them less efficient in colonizing new habitats comparing to angiosperms in this region (Elias 2013), and gymnosperm seed size and dispersal syndromes do not appear to trade‐off with shade tolerance as seen in angiosperms do (Hewitt 1998). Second, gymnosperms can mostly only compete with established angiosperms in certain climatic settings (Bond 1989). For example, *Picea critchfieldii*, a once‐dominant species in the Lower Mississippi Valley, went extinct, possibly due to competition from angiosperm trees invading in response to post‐LGM warming (Jackson and Weng 1999). Boreal tree species appear to trade off between cold tolerance and growth rate, so at their southern range limits they are out‐competed by fast‐growing species (Loehle 1998). This may partly explain the negative effect of postglacial tree accessibility on gymnosperm phylogenetic diversity and endemism in the less glaciated East forest region. Third, due to their long‐branched phylogeny and niche conservatism, ecological function in gymnosperms may reflect adaptation to deeper‐time climate change. As most of the present genera originated in the Miocene aridification, survivors of drought may have been physiologically preadapted and rediversified in the later colder climate episodes (Crisp and Cook 2011), causing gymnosperm PE to be more sensitive to water relations than angiosperms and increased with PRS. Last, despite recent diversification, overall diversity of gymnosperms is much lower than that of angiosperms, creating more stochastic phylogenetic clustering patterns from assemblage of fewer species, for example, where Pinaceae and Cupressaceae failed to co‐occur would show strong clustering.

We note that postglacial tree accessibility measure could not easily be disentangled from the current temperature in this study region, and its effect may therefore have been underestimated. Postglacial migration lag might be better represented with a more detailed accessibility measure, for example, by mapping each individual species (Normand et al. 2011), which might help disentangle its effects from those of current temperature. Also, the current ACC measure is based on the overall estimated distribution of forests in LGM, which has limited capability of capturing dynamics of subtropical and tropical trees that likely did not survive close to the LGM forest limits, so our ACC is more applicable to cold‐tolerant gymnosperms and angiosperms.

In conclusion, current climate provides the strongest explanatory power for the geographic variation in phylogenetic assemblage structure in trees across North America. This pattern was consistent with previous studies of angiosperm trees. Still, current climate did not as clearly dominate the patterns in gymnosperms. Furthermore, glacial–interglacial climate stability appeared to also have strong influences: The relatively stable regions in North America hosted more phylogenetic endemism and diversity, as well as exhibiting a more phylogenetically clustered pattern, the latter likely due to unique clades with either relatively recent diversification, perhaps coupled to separate preservation in different refugia. These results demonstrate the supplementary importance of paleoclimatic factors in shaping biogeographic patterns (Svenning et al. 2015), perhaps particularly so in ancient clades. Gymnosperm trees, dominating vast forests in North America and the Holarctic realm, deserve more special focus in future‐related studies, given that their patterns were less fully explained.

## Conflict of Interest

None declared.

## Supporting information


**Table S1.** List of angiosperm synonyms involved in the names used in the Atlas of United States Trees (Little 1971–1978) according to The Plant List.

**Data S1.** Newick format phylogenetic tree of angiosperm species in this study.

**Data S2.** Newick format phylogenetic tree of gymnosperm species in this study.
