# Supplementary material for: Phylogenetic assemblage structure of North American trees is more strongly shaped by glacial–interglacial climate variability in gymnosperms than in angiosperms
Source: Ecol Evol. 2016 Apr 3;6(10):3092–106. doi: 10.1002/ece3.2100 (PMC4870196; doi:10.1002/ece3.2100)
Supplement: Supplementary file 1 — Table S1. List of angiosperm synonyms involved in the names used in the Atlas of United States Trees (Little 1971–1978) according to The Plant List. [file ECE3-6-3092-s001.docx]

**Supplementary Materials**

**S1 Table:** List of angiosperm synonyms involved in the names used in the Atlas of United States Trees (Little 1971-1978) according to The Plant List. In this list and the resulting phylogeny, original names in the Atlas of United States Trees were replaced by their counterpart in the accepted names of this study.

| **Original** | **Accepted** |
| --- | --- |
| *Acer barbatum* | *Acer glabrum* |
| *Acer grandidentatum* | *Acer saccharum* |
| *Acer leucoderme* | *Acer saccharum* |
| *Acer nigrum* | *Acer saccharum* |
| *Aesculus octandra* | *Aesculus flava* |
| *Alnus rugosa* | *Alnus incana* |
| *Alnus sinuata* | *Alnus alnobetula* |
| *Alnus tenuifolia* | *Alnus incana* |
| *Arbutus texana* | *Arbutus xalapensis* |
| *Ardisia escallonoides* | *Ardisia escallonioides* |
| *Betula uber* | *Betula lenta* |
| *Bumelia celastrina* | *Sideroxylon celastrinum* |
| *Bumelia lanuginosa* | *Sideroxylon lanuginosum* |
| *Bumelia lycioides* | *Sideroxylon lycioides* |
| *Bumelia tenax* | *Sideroxylon tenax* |
| *Capparis cynophallophora* | *Quadrella cynophallophora* |
| *Capparis flexuosa* | *Cynophalla flexuosa* |
| *Carya tomentosa* | *Carya alba* |
| *Castanea alnifolia* | *Castanea pumila* |
| *Castanopsis chrysophylla* | *Chrysolepis chrysophylla* |
| *Celtis reticulata* | *Celtis laevigata* |
| *Cercocarpus betuloides* | *Cercocarpus montanus* |
| *Cercocarpus breviflorus* | *Cercocarpus montanus* |
| *Cereus giganteus* | *Carnegiea gigantea* |
| *Citharexylum fruticosum* | *Citharexylum spinosum* |
| *Cornus occidentalis* | *Cornus sericea* |
| *Cornus stolonifera* | *Cornus sericea* |
| *Cornus stricta* | *Cornus foemina* |
| *Cowania mexicana* | *Purshia mexicana* |
| *Dalea spinosa* | *Psorothamnus spinosus* |
| *Dipholis salicifolia* | *Sideroxylon salicifolium* |
| *Genipa clusiifolia* | *Casasia clusiifolia* |
| *Gymnocladus dioicus* | *Gymnocladus dioica* |
| *Halesia parviflora* | *Halesia carolina* |
| *Lithocarpus densiflorus* | *Notholithocarpus densiflorus* |
| *Magnolia ashei* | *Magnolia macrophylla* |
| *Magnolia pyramidata* | *Magnolia fraseri* |
| *Malus diversifolia* | *Malus fusca* |
| *Manilkara bahamensis* | *Manilkara jaimiqui* |
| *Mastichodendron foetidissimum* | *Sideroxylon foetidissimum* |
| *Morus microphylla* | *Morus celtidifolia* |
| *Myrica cerifera* | *Morella cerifera* |
| *Opuntia fulgida* | *Cylindropuntia fulgida* |
| *Photinia arbutifolia* | *Heteromeles arbutifolia* |
| *Pinckneya pubens* | *Pinckneya bracteata* |
| *Pithecellobium flexicaule* | *Ebenopsis ebano* |
| *Pithecellobium guadalupense* | *Pithecellobium keyense* |
| *Pithecellobium pallens* | *Havardia pallens* |
| *Populus arizonica* | *Populus fremontii* |
| *Psidium longipes* | *Mosiera longipes* |
| *Quercus dunnii* | *Quercus palmeri* |
| *Quercus durandii* | *Quercus sinuata* |
| *Quercus graciliformis* | *Quercus canbyi* |
| *Quercus nuttallii* | *Quercus texana* |
| *Quercus prinus* | *Quercus michauxii* |
| *Rapanea punctata* | *Myrsine cubana* |
| *Rhamnus californica* | *Frangula californica* |
| *Rhamnus purshiana* | *Frangula purshiana* |
| *Rhus copallina* | *Rhus copallinum* |
| *Rhus laurina* | *Malosma laurina* |
| *Robinia kelseyi* | *Robinia hispida* |
| *Roystonea elata* | *Roystonea regia* |
| *Salix fluviatilis* | *Salix melanopsis* |
| *Salix hindsiana* | *Salix exigua* |
| *Salix mackenzieana* | *Salix prolixa* |
| *Sambucus mexicana* | *Sambucus canadensis* |
| *Sapium biloculare* | *Sebastiania bilocularis* |
| *Simarouba glauca* | *Simarouba amara* |
| *Styrax americana* | *Styrax americanus* |
| *Styrax grandifolia* | *Styrax grandifolius* |
| *Styrax platanifolia* | *Styrax platanifolius* |
| *Thrinax morrisii* | *Leucothrinax morrisii* |
| *Tilia heterophylla* | *Tilia americana* |
| *Vauquelinia pauciflora* | *Vauquelinia californica* |
| *Yucca mohavensis* | *Yucca schidigera* |
| *Yucca torreyi* | *Yucca faxoniana* |
| *Zanthoxylum hirsutum* | *Zanthoxylum clava-herculis* |
